# Frequency dispersion reveals chromophore diversity and colour-tuning mechanism in parrot feathers

**DOI:** 10.1098/rsos.172010

**Published:** 2018-07-04

**Authors:** Jonathan E. Barnsley, Elliot J. Tay, Keith C. Gordon, Daniel B. Thomas

**Affiliations:** 1 Department of Chemistry, University of Otago, Dunedin, New Zealand; 2 The Dodd-Walls Centre, University of Otago, Dunedin, New Zealand; 3 Institute of Natural and Mathematical Sciences, Massey University, Auckland, New Zealand

**Keywords:** frequency dispersion, museum specimens, pigment, plumage, Psittaciformes, Raman spectroscopy

## Abstract

Variation in animal coloration is often viewed as the result of chemically distinct pigments conferring different hues. The role of molecular environment on hue tends to be overlooked as analyses are mostly performed on free pigments extracted from the integument. Here we analysed psittacofulvin pigments within parrot feathers to explore whether the *in situ* organization of pigments may have an effect on hue. Resonance Raman spectra from a red region of a yellow-naped amazon *Amazona auropalliata* tail feather show frequency dispersion, a phenomenon that is related to the presence of a range of molecular conformations (and multiple chromophores) in the pigment, whereas spectra from a yellow region on the same feather do not show the same evidence for multiple chromophores. Our findings are consistent with non-isomeric psittacofulvin pigments behaving as a single chromophore in yellow feather barbs, which implies that psittacofulvins are dispersed into a structurally disordered mixture in yellow feathers compared with red feathers. Frequency dispersion in red barbs may instead indicate that pigments are structurally organized through molecule–molecule interactions. Major differences in the hues of parrot feathers are thus associated with differences in the organization of pigments within feathers.

## 1. Introduction

Animal coloration covers the full visual gamut and is achieved through a chemically diverse group of pigments [1]. Colours and patterns in skin, scales and feathers can closely match an environmental background to help an animal evade predators, or alternatively, to help predators ambush prey [2]. Bright colours that poorly match environmental backgrounds can be intentionally conspicuous so as to advertise danger to potential predators, or be used to mimic the colours of dangerous animals [3], and might also advertise the quality of a potential mate [4]. Irrespective of colour palette and function, integument colours are likely under strong selection pressures, and these pressures provide an exciting context in which to consider novel pigmentation systems [5]. What advantages must new pigments offer when they first emerge if they are to be selected? Here we focus on the pigment system in the plumages of parrots (Psittaciformes) to test for evidence of tuneable coloration as a potential novelty over carotenoid-based plumage colours [6].

Pink, red, orange and yellow feather colours in most birds are conferred by carotenoid pigments (e.g. canaries, flamingos), which are extracted from the diet and concentrated at the base of growing feathers [7]. The equivalent colours in parrot feathers are instead conferred by pigments that are unique to parrots [5,8] (at least among birds [9]). Parrots were first reported to have chemically distinct ‘psittacofulvin’ plumage pigments in 1882 [5]. More recent analyses have shown that psittacofulvin pigments are polyenal compounds that differ from carotenoids by lacking methyl side groups and ionone rings [8,10]. A set of structurally similar psittacofulvin compounds are responsible for the red colours of distantly related parrots [11,12]. A recent study provided evidence that yellow feathers from a budgerigar *Melopsittacus undulatus* contained almost the same composition of psittacofulvin-type pigments as are found in red feathers, and proposed that the colour shift from red to yellow might be attributed to the absence in yellow feathers of the psittacofulvin pigment with the longest conjugated carbon backbone [13]. Here we explore whether colour variation in parrot feathers may also or instead be attributed to differences in the structural organization of psittacofulvin pigments in feathers. We are presenting a fourth option to the observation made by Cooke *et al.* [13] that ‘ … the visual difference between red and yellow parrot feathers is most likely due to presence or absence of the C_20_ pigment component, a different oxidation state at the end of the polyene acyl chains, or both … ’

Stradi and co-workers [8] observed that the pigments in red plumage appeared orange after being extracted from the feather, and proposed that the colour shift may result from molecule–molecule interactions causing a conformational change (i.e. ‘ … bathochromic effect may be of a supramolecular nature … ’). The classic example of a molecular conformation model for animal coloration is the carotenoid–protein complex that occurs in crustaceans (crustacyanin [14]). The usually red astaxanthin carotenoid is distorted into a new conformation as it interacts with the protein component of crustacyanin [15]: the interaction alters the electronic structure along the conjugated backbone and in turn alters how the pigment molecule interacts with visible light. For psittacofulvin-pigmented feathers, we further observe that they are unlike carotenoid-pigmented feathers in that yellow and red pigments can occur in the same feather, often associated with a colour gradient, indicating that plumage coloration is manipulated during feather growth. Here we use an *in situ* method to collect information about the composition and conformation of psittacofulvin pigments. With Raman spectroscopy, we seek (1) confirmation that psittacofulvins are responsible for the full range of magenta to yellow parrot feather hues and (2) evidence that there are substantial differences in the molecular environments in red versus yellow feather barbs.

Raman spectroscopy is an analytical technique that provides information about molecular composition and molecular environments by analysing light that scatters from a sample. Raman spectroscopy becomes resonance Raman spectroscopy when the wavelength of incident light coincides with a wavelength that is strongly absorbed by the sample (i.e. excitation energy matches electronic transition) [16,17]. Resonance with an electronic transition typically results in a 10^5^ increase of Raman intensity, allowing the scattering from specific components in a mixed sample to be enhanced [18]. Information about the diversity of chromophores (i.e. structural and conformational isomers) in a sample can, therefore, be revealed with resonance Raman spectroscopy by using a number of excitation wavelengths to selectively enhance each chromophore in the mixed sample [18]. This technique has been widely used in polymer science to identify molecule–molecule interactions and molecular conformations [19–21], and is particularly advantageous for studying coloured tissues. The signal enhancement from resonance Raman spectroscopy provides a major advantage as the quantity of a conjugated dye present in tissue is can be very low (e.g. less than 1 mg g^−1^ for parrot feathers) [12]. Most importantly, Raman spectroscopy has the ability to detect molecular interactions that only exist *in situ* (in contrast to *ex situ* analysis techniques, e.g. liquid chromatography, mass spectrometry).

Testing for molecular environmental differences as an explanation for colour variation provided two aims for our study. First, we sought to confirm that the entire range of magenta to yellow hues in parrot feathers were underpinned by the same basic group of psittacofulvin pigments identified by Stradi and co-workers [8]. If differences in molecular conformation are responsible for the magenta to yellow gamut in parrot feathers, then we should find evidence for the same pigments in parrot feathers irrespective of feather hue. Stradi and co-workers [8] proposed that scarlet macaw feathers contain pigments with conjugated carbon backbones (14 to 20 carbon atoms long), and Veronelli and co-workers [10] showed that these pigments produce a simple Raman spectrum with two main peaks [8]. McGraw & Nogare [12] reported that red feathers from distantly related parrot species all contained a mixture of the same polyenal compounds. Cooke *et al.* [13] reported that the C_14_, C_16_ and C_18_ length psittacofulvins found in red feathers also occur in the yellow feathers of budgerigars. We hypothesize that parrot feathers across the magenta–red–orange–yellow colour range for psittacofulvins will produce the same simple Raman spectrum [10,12] with shifts in the peak position arising from variation in backbone conformation. Our hypothesis is inversely based on observations from carotenoid-pigmented plumage [22]. The pink, purple, red, orange and yellow colours in most feathers are conferred by different carotenoid compounds that show a range of functional groups and backbone bonding configurations [7]. Raman spectra from carotenoid-pigmented feathers often feature novel spectral peaks due to the characteristic functional groups [22]. We would not expect to see evidence for additional functional groups in Raman spectra from different coloured parrot feathers if all magenta, red, orange and yellow colours in parrot feathers are produced by the same suite of pigments.

Our second aim was to survey yellow and red feather barbs to ascertain the nature of the chromophores that provide colour. By using resonance Raman spectroscopy, in which the excitation wavelength is varied, it is possible to probe the presence of a single chromophore or multiple chromophores [17]. Multiple chromophores will exist for a molecule that has a distribution of possible conformations, with each chromophore having different lengths of conjugation, or different degrees of communication between adjacent pigment molecules that in turn extends the effective conjugation length. For an extended conjugated system (i.e. a communicating network of adjacent chromophores) the vibrational frequencies observed in Raman spectra will be responsive to the effective conjugation length. Thus, if the effective length is shortened because of conformational twisting, the vibrational frequency will be higher. If the conjugation length is instead extended through intermolecular interactions, then the frequency of the vibrational modes will be lower. Extended conjugation leads to a lower energy chromophore, hence this conformation is seen as red excitation wavelengths, whereas short effective conjugation length chromophores are seen with blue laser excitation. As a result, the frequency of the Raman spectral bands shifts as a function of excitation wavelength—this is called frequency dispersion [23–25]. If there is only one type of chromophore present, then no frequency dispersion is observed. Our analytical approach is based on analogous investigations into molecule–molecule interactions in synthetic π-conjugated systems, such as polythiophene and polyacetylene systems [26,27].

## 2. Material and methods

### 2.1. Feathers

Feathers analysed in this study were from the collections of the Division of Birds, National Museum of Natural History, Smithsonian Institution (NMNH; Washington DC, USA), and the collections of Massey University (Auckland campus, New Zealand). Feathers from NMNH had been collected prior to the commencement of this research and were either part of a study skin specimen or had been plucked and stored by museum staff in the process of making skeleton specimens. A total of 26 feathers from parrots in the NMNH collections were analysed (appendix A). One feather from a red-footed booby *Sula sula* (also NMNH) was used for spectral comparison. The red-footed booby feather and several of the parrot feathers were part of the consumptive feather file in the Division of Birds, NMNH.

Moulted feathers from a yellow-naped amazon *Amazona auropalliata* were donated to Massey University in September 2017. One tail feather from the donated collection was analysed at the Department of Chemistry, University of Otago (Dunedin, New Zealand).

### 2.2. Psittacofulvin colour gamut

The 26 NMNH parrot feathers each featured a region of barbs made up of predominantly one colour (e.g. magenta; appendix A). The coloured regions of these parrot feathers were analysed on site in the collections of the Division of Birds, NMNH. Three spectra were collected from nearby positions on each feather using a Raman microscope (Nomadic, BaySpec, San Jose CA, USA) that had a 1064 nm excitation Nd:YAG laser with a 512 pixel InGaAs array detector for a maximum spectral range of 277–1886 cm^−1^ (electronic supplementary material, tables S1 and S2). A 40× microscope objective (EPlan, Nikon Instruments, Tokyo, Japan) with a 25 µm confocal pinhole produced a minimum spot size diameter of 2 µm. A Raman spectrum from a white wing feather of a red-footed booby was collected as a β-keratin spectral standard (electronic supplementary material, table S1). Triplicate Raman spectra from each feather were preprocessed by first reducing the spectral range to 900–1800 cm^−1^, followed by Savitzky–Golay filtering (sgolay::signal 0.7–6) [28] and baseline correction (baseline::baseline 1.2-1) [29] in R v. 3.4.1 [30]. Triplicate preprocessed spectra were then intensity normalized (i.e. intensities scaled between 0 and 1) and averaged (electronic supplementary material, table S2). Peak parameters were calculated using Spectragryph 1.2 [31].

Raman instruments that produced higher resolution spectra over larger wavenumber ranges were used to provide a second spectral dataset from five of the 26 NMNH parrot feathers (electronic supplementary material, table S3). This second spectral dataset was produced to support peak assignments made for the 26 feather dataset, and was based on feathers that could be transported out of the Division of Birds to two additional Raman instruments. Spectra were collected with a Nicolet Almega XR spectrometer (Thermo Electron Corporation, Madison, WI, USA) using 780 nm excitation to produce spectra across 100–3500 cm^−1^ at 3 cm^−1^ resolution, and an NXR FT-Raman module coupled to a 6700 FTIR spectrometer (Thermo Electron Corporation) which used 1064 nm excitation to produce spectra across 100–3700 cm^−1^ at 4 cm^−1^ resolution.

### 2.3. Resonance Raman experiment

**Figure 1.**
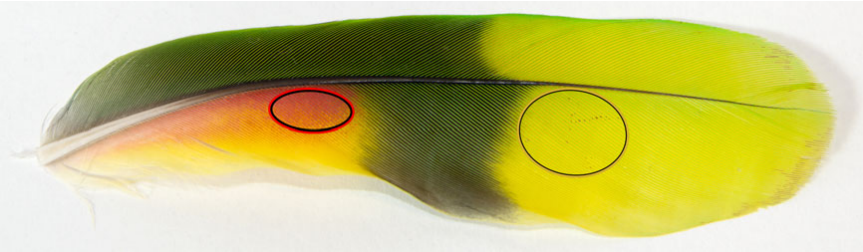
Resonance Raman spectra were collected from a red region and from a yellow region (circled) on a tail feather from a yellow-naped amazon parrot *A. auropalliata*. Feather is approximately 10 cm long.

Resonance Raman spectra were collected from a yellow region and from a red region on a single tail feather from a yellow-naped amazon parrot (figure 1; electronic supplementary material, table S4). The resonance Raman instrument consisted of an excitation beam with a collection lens in a 135° backscattering arrangement [32–34]. Scattered photons were focused on the entrance slit of an Acton Isoplane 320 spectrograph with a 1200 grooves/mm grating (Princeton Instruments, Trenton, NJ, USA), which dispersed the radiation in a horizontal plane on a PyLoN 400BR liquid-nitrogen-cooled CCD detector (Princeton Instruments). An Innova I-302 krypton ion laser (Coherent Inc., Santa Clara, CA, USA) was used to provide excitation wavelengths of 351, 407, 413, 568 and 648 nm; CrystaLaser solid-state units were used for 375, 448, 532 and 594 nm (CrystaLaser, Reno, NV, USA) while cobolt solid-state diodes provided the 458, 491, 515 nm wavelengths (Cobolt Group Inc., Solna, Sweden). Notch filters (Kaiser Optical Inc., Ann Arbour, MI, USA) or long-pass filters (Semrock Inc., Rochester, NY, USA) matched to these wavelengths were used to remove the laser excitation line. The 594 nm laser required an additional 633 nm filter to remove an unwanted laser line. The acquisition windows were calibrated to within 1 pixel (which corresponds to less than 3 cm^−1^ at 351 to 413 nm and 1 cm^−1^ or better for longer excitation wavelengths) using a 1 : 1 volume mixture of acetonitrile and toluene, which provides reference peaks over the spectral range [35]. Taking into account spectral resolution (which ranges from less than 3 cm^−1^ to lower values with red excitation wavelengths) and calibration resolution, spectral variations greater than 3 cm^−1^ are considered to be sample-related. Power at the feather sample was maintained at approximately 2 mW to avoid sample burning. Data from three spot locations were collected with a spot size around 1 mm, which were then averaged to reduce subsampling issues.

A 785 nm wavelength was obtained using an Alpha 300R Raman microscope and a UHTS300S spectrometer implementing a 600 grooves.mm^−1^ grating to disperse signal to collect on a DU401 BR,DD CCD with an integration time of 0.05 s (WiTec GmbH, Ulm, Germany). Image scans used 50 mW of power without sample burning (due to non-resonance and short dwell time), 150 × 150 data points were sampled at 30 × 30 µm resolution, and a 10× objective magnification and with dwell time of 0.1 s. Data points were accumulated to provide a non-resonant spectrum with reduced subsampling issues.

### 2.4. Fluorescence and pigment distribution

Previous studies have observed that yellow parrot feathers can fluoresce [36]. Here the opportunity was taken to visualize the spatial distribution of emission across small regions of the yellow-naped amazon feather. Using the WiTec 300R Raman microscope mentioned above, both bright-field and Raman maps of barbs in the red and yellow regions were gathered. In this case, excitation of 532 nm was used due to the advantageous increase of the signal-to-noise ratio (SNR) when a low laser power is used (approx. 2 mW). Of note, 150 × 150 data points were sampled at 170 × 170 µm resolution using 10× objective magnification. Integration time was decreased to 0.023 s. No emission or baseline correction has been applied to the dataset. Raman maps were generated using the WiTec Control/Project 4.1 (WiTec GmbH, Ulm, Germany) software, and employed ±50 cm^−1^ sum filters at approximately 1140 and approximately 1540 cm^−1^.

### 2.5. UV–vis absorption

UV–vis spectra from several coloured regions on the yellow-naped amazon feather were measured using a Perkin Elmer Lambda 950 spectrophotometer equipped with a 150 mm integrating sphere. This system used a tungsten–halogen lamp, a deuterium lamp and a R6872 photomultiplier. A 1 nm step size was used to scan from 300 to 800 nm.

### 2.6 Frequency dispersion

**Figure 2.**
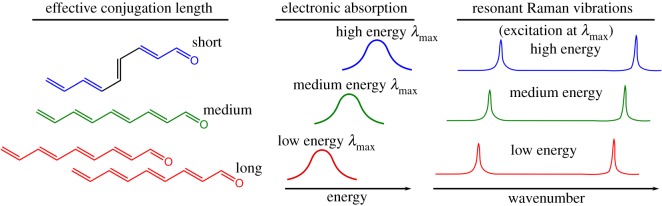
An illustration of frequency dispersion as described by the AMM.

As described in the introduction, frequency dispersion is the shifting of Raman bands as a function of excitation wavelength because of the presence of a distribution of chromophores of differing conjugation length. In these samples, the strongest Raman bands lie between 1130 and 1145 cm^−1^, and 1525 and 1550 cm^−1^. These peaks are due to the C–C and C=C stretching modes, respectively [37], along the conjugated carbon backbone of the psittacofulvin pigment [10]. The wavenumber values for these peaks are labelled $\tilde{\nu }(\text{C−C})$ and $\tilde{\nu }(\text{C=C})$ (appendix B). The frequency dispersion was quantified using the amplitude mode model (AMM) [23]. The AMM describes distortion in π-conjugated molecules as the relationship between vibrational energy and excitation energy (figure 2). The model calculates a ratio between the wavenumber of a vibrational mode obtained with the lowest excitation energy used (${\tilde{\nu }}_{0}^{R}$), and the vibrational wavenumbers of the same mode obtained using other excitation energies (${\tilde{\nu }}_{n}^{R}$). The product of this ratio for all vibrational modes undergoing frequency dispersion is used to calculate the effective electron–phonon coupling constant ($\bar{\lambda }$) (‘product rule’: equation (2.1)). A dispersion rate parameter (***D***) is then calculated as the slope of the relationship between the effective electron–phonon coupling constant and excitation energy (***E_L_***) (equation 2).

This dispersion rate parameter relates to the co-cooperativity of adjacent molecules. For example, greater ***D*** values (greater frequency dispersion) are found in conducting polymers which are highly coupled between neighbouring molecules [26,27]. These materials have planar arrangements with long conjugation lengths due to these π–π interactions. On the contrary, materials which do not have strong interactions between adjacent molecules tend not to exhibit frequency dispersion and have a ***D*** value close to zero [23–25].

2.1 $$\prod_{n}{\left(\frac{{\tilde{\nu }}_{n}^{R}}{{\tilde{\nu }}_{0}^{R}}\right)}^{2}=\text{2}\bar{\lambda }$$

and

2.2 $$D=\frac{\Delta \left({\prod_{n}\left({\tilde{\nu }}_{n}^{R}/{\tilde{\nu }}_{0}^{R}\right)}^{2}\right)}{\Delta E_{L}}.$$

## 3. Results

### 3.1. Psittacofulvin colour gamut

**Figure 3.**
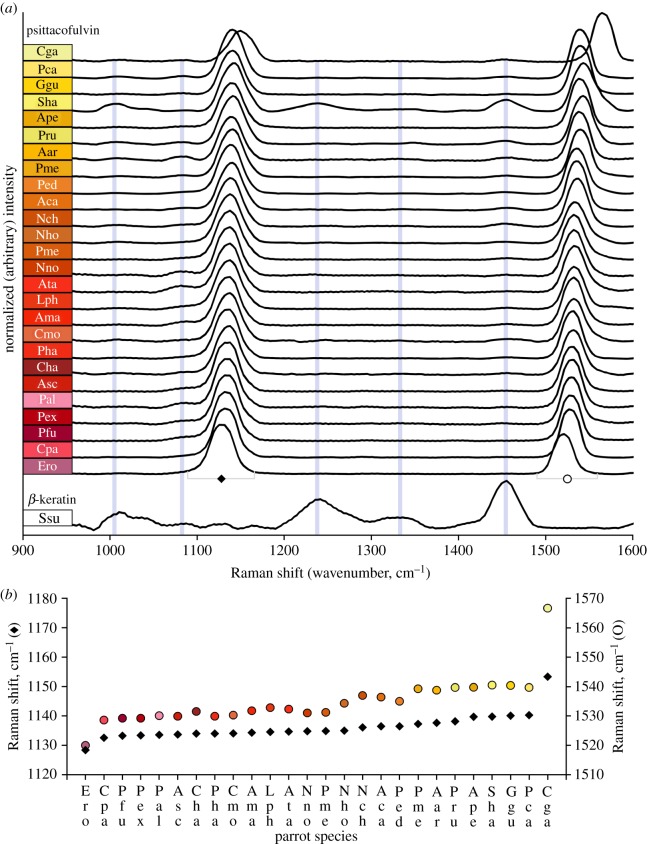
(*a*) Raman spectra from 26 parrot feathers show two major peaks ($\tilde{\nu }(\text{C−C})$ closed diamond; $\tilde{\nu }(\text{C=C})$ open circle) attributed to psittacofulvin pigments [10]. A spectrum from a white wing feather shows only evidence for unpigmented β-keratin. Some Raman spectra from parrot feathers show both psittacofulvin and β-keratin peaks. (*b*) Peak positions for $\tilde{\nu }(\text{C−C})$ and $\tilde{\nu }(\text{C=C})$ are higher in spectra from yellow feathers compared with peak positions in spectra from orange, red and magenta feathers. Ssu, *Sula sula*. See appendix A for parrot species abbreviations.

All Raman spectra from feathers in the 26 parrot (1064 nm excitation) dataset featured major peaks around 1130 cm^−1^ and 1500 cm^−1^ (figure 3*a*), and both peaks varied in position (figure 3*b*). $\tilde{\nu }(\text{C}-\text{C})$ and $\tilde{\nu }(\text{C=C})$ values tended to increase in more ‘blue-shifted’ feather colours (i.e. magenta > red > orange > yellow) (figure 3*b*). All additional Raman spectral peaks were attributed to β-keratin: 1006 cm^−1^ phenylalanine, 1240 cm^−1^ amide I, 1450 cm^−1^ CH_2_ groups and 1670 cm^−1^ amide III [38]. Hence, no evidence for chromophore functionality beyond a conjugated carbon backbone was found in spectra from the 26 parrot feathers.

Raman spectra collected with 532 and 780 nm excitation showed two additional peaks at approximately 2× the value of $\tilde{\nu }(\text{C−C})$ and $\tilde{\nu }(\text{C=C})$ in each spectrum. The positions of these peaks were consistent with the first overtones for the fundamental transitions [39].

UV–vis spectra for the yellow, black and red regions show strong absorption from 350 to 600 nm (electronic supplementary material, figure S1). The black and red spectra also show a low energy absorption around 650 nm. The absorption profiles of the red and yellow regions provide satisfactory overlaps with available excitation wavelengths (351–647 nm) for resonance Raman measurements below.

### 3.2. Resonance Raman experiment

**Figure 4.**
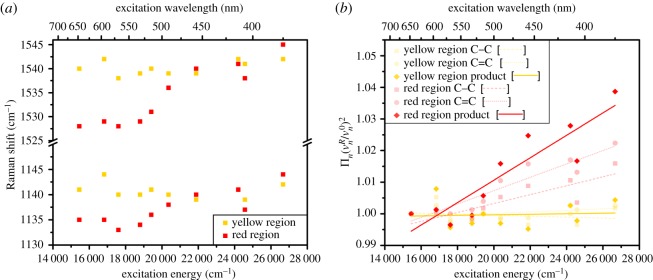
Raman spectral data from a yellow region and a red region on a yellow-naped amazon *A. auropalliata* tail feather. (*a*) Raman shifts in $\tilde{\nu }(\text{C−C})$ (1130–1145 cm^−1^) and in $\tilde{\nu }(\text{C=C})$ (1525–1545 cm^−1^) when excited with different wavelengths. Spectra from the red region of the feather show substantial shifts in peak position with excitation with different wavelengths. (*b*) $\prod_{n}{\left(\nu_{n}^{R}/\nu_{n}^{0}\right)}^{2}$ increases with excitation energy for $\tilde{\nu }(\text{C−C})$ and $\tilde{\nu }(\text{C=C})$ bands in the red feather region, but not the yellow feather region. Excitations at 351 nm, 448 nm and 785 nm have been excluded from this analysis.

Resonance Raman spectra from the yellow-naped amazon parrot feather showed the expected peaks for psittacofulvin pigments (electronic supplementary material, tables S4 and S5). SNRs in spectra from the yellow area of the feather were lower than SNRs in spectra from the red area due to an emission background in yellow-area spectra (determined from visual inspection). Irrespectively, $\tilde{\nu }(\text{C−C})$ and $\tilde{\nu }(\text{C=C})$ values in yellow-area spectra were well defined across the range of excitation wavelengths. The spectrum collected at 448 nm produced spurious results and was removed from further analysis (electronic supplementary material, table S5). Likewise, data collected at 351 and 785 nm weakened the relationships between excitation energy and $\tilde{\nu }(\text{C−C})$ and $\tilde{\nu }(\text{C=C})$, likely because red psittacofulvins are pre-resonant at these wavelengths, and hence these data were excluded from dispersion rate analyses. $\tilde{\nu }(\text{C−C})$ and $\tilde{\nu }(\text{C=C})$ values in spectra from the yellow feather area showed 5 and 6 cm^−1^ ranges, respectively, across all excitation wavelengths. By contrast, variations in $\tilde{\nu }(\text{C−C})$ and $\tilde{\nu }(\text{C=C})$ from the red feather area were 11 cm^−1^ and 17 cm^−1^, respectively (figure 4*a*). Strong positive correlations were consequently found between the ${\left({\tilde{\nu }}_{n}^{R}/{\tilde{\nu }}_{0}^{R}\right)}^{2}$ values and the excitation energy (cm^−1^) values in data from the red feather region: $\tilde{\nu }(\text{C−C})$ *R*^2^ = 0.680, *p*-value = 0.002; $\tilde{\nu }(\text{C=C})$ *R*^2 ^= 0.872, *p*-value < 0.001 (figure 4*b*). A strong positive relationship was also found between $2\bar{\lambda }$ and excitation energy (cm^−1^) in data from the red feather region (*R*^2^ = 0.806, *p*-value = 0.002). The dispersion rate parameter ***D*** for the red feather region was 3.55 × 10^−6^ cm^−2^ (2.86 × 10^−2^ eV^−1^) with a model fit error of 16%. Meaningful relationships between ${\left({\tilde{\nu }}_{n}^{R}/{\tilde{\nu }}_{0}^{R}\right)}^{2}$ values and excitation energies were not found for the yellow feather region: $\tilde{\nu }(\text{C−C})$ *R*^2^ = 0.071, *p*-value = 0.546; $\tilde{\nu }(\text{C=C})$ *R*^2 ^= 0.115, *p*-value = 0.179 (figure 4*b*). No meaningful relationship was, therefore, found between $2\bar{\lambda }$ and excitation energy (cm^−1^) in data from the yellow feather region (*R*^2^ = 0.119, *p*-value = 0.002). The dispersion rate parameter ***D*** for the yellow feather region (8.47 × 10^−8^ cm; 6.97 × 10^−4^ eV^−1^) was much lower than for the red feather region and the model fit error (158%) was much higher.

**Figure 5.**
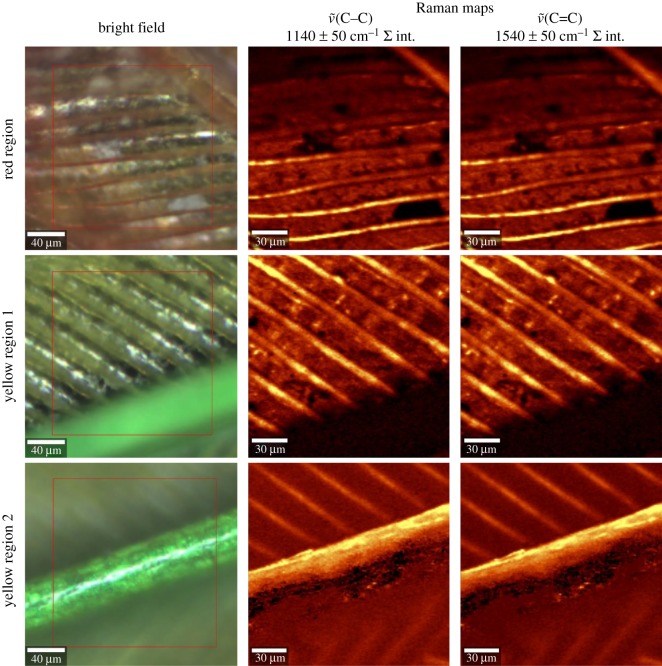
Bright-field and 532 nm excitation mapping of three regions on a yellow-naped amazon *A. auropalliata* tail feather. Raman maps show the sum of intensity values from Raman spectral peaks at 1090 to 1190 cm^−1^ and 1490 to 1590 cm^−1^, corresponding to the $\tilde{\nu }(\text{C−C})$ and $\tilde{\nu }(\text{C=C})$ modes, respectively. Bright colours show high emission.

Bright-field images indicate that emission is present in the yellow feather region, as has been observed in the literature, [36] while the red region appears to be non-emissive (figure 5). $\tilde{\nu }(\text{C−C})$ and $\tilde{\nu }(\text{C=C})$ bands are observed from the emissive barbs which, considering the emission background, indicate substantial psittacofulvin concentration present. While these data cannot unequivocally identify psittacofulvins as the active emissive material (instead of β-keratin or some unidentified third component), Ozaki *et al.* identified a correlation between ***D*** and emissive characteristics in a group of π-conjugated polymers (e.g. poly(thienylene vinylene)), which is consistent with those seen here [40]. Materials with low Raman dispersion were emissive (yellow region), while high Raman dispersion was observed in materials with non-emissive properties (red region). Both absorption and emission characteristics of psittacofulvins are expected to be perturbed by conformational parameters; the former has been well investigated and elaborated on here, while the latter requires further investigation.

## 4. Discussion

### 4.1. Aim 1: psittacofulvin colour gamut

Magenta, red, orange and yellow feathers from parrots produce the same simple Raman spectrum at 1064 nm excitation, with the only difference between spectra being the wavenumber values of the two peaks that identify the conjugated carbon backbone, i.e. $\tilde{\nu }(\text{C−C})$ and $\tilde{\nu }(\text{C=C})$. No evidence for additional functional groups was detected in this study. Hence, the only differences between psittacofulvin chromophores conferring different hues are systematic shifts in the effective conjugation lengths of these compounds. We did not detect evidence for a carbonyl group in spectra from any feathers; Stradi and co-workers [8] reported that psittacofulvins from red feathers were aldehydes, and Cooke *et al.* [13] reported an absence of aldehyde functionality in psittacofulvins from yellow feathers. McGraw & Nogare [12] had previously shown that variation in hue across red feathers corresponds with total pigment concentrations, and Stradi and co-workers [8] had proposed that the red to orange colour shift in extracted psittacofulvins ‘…could be of supramolecular nature … ’ The gradual shift in $\tilde{\nu }(\text{C−C})$ and $\tilde{\nu }(\text{C=C})$ from magenta to yellow feathers helps confirm that psittacofulvins are a broadly tuneable plumage system. The presence of the C_20_ psittacofulvin [13] is associated with major shifts in hue and is potentially important to the structural organization of pigments in red feather barbs (elaborated below).

### 4.2. Aim 2: matrix tuning in pigment coloration

Raman spectra were collected from the red region of a yellow-naped amazon parrot feather and showed wavelength-dependent shifts in peak position (up to 17 cm^−1^). These major shifts indicate the presence of multiple chromophores that may represent non-isomeric pigments or conformational isomers. The presence of multiple chromophores was strongly supported by the significant positive relationship between $\text{2}\bar{\lambda }$ and excitation energy [23,41,42]. Frequency dispersion effects have previously been associated with polymer–polymer or oligomer–oligomer interactions. As described above, closely arranged conformational isomers can distort one another's structure, which changes the distribution of electrons around each chromophore and in turn alters the energy gap between ground and excited electronic states [43]. Bonding energies are likewise affected, which manifest as shifts in the position of peaks in vibrational frequencies [43]. The frequency dispersion observed here could equally be identifying the multiple psittacofulvin compounds without inter-molecular associations [8,12]. Hence, based on our promising data alone, we cannot definitively state that the frequency dispersion we observe is driven by molecule–molecule interactions. Consider though that frequency dispersion is absent in yellow feathers despite the expected presence of multiple non-isomeric psittacofulvin compounds [13].

The ***D*** value of 2.86 × 10^−2^ eV^−1^ for the red feather region was comparable to that found for sterically separated polymers, where some π–π interactions of neighbouring units are present (e.g. poly(*p*-phenylene vinylene)) [27]. However, the ***D*** value is lower than the structurally analogous *trans*-polyacetylene (***D*** values ≥ 5.0 × 10^−2^ eV^−1^), which is well known for strong π–π interactions [27].

Raman spectra from the yellow region of a yellow-naped amazon parrot feather showed only minor variation in $\tilde{\nu }(\text{C−C})$ and $\tilde{\nu }(\text{C=C})$ values and no substantial evidence for multiple chromophores. The non-isomeric mixture of psittacofulvin pigments, therefore, behaves as a single chromophore in yellow feather barbs, which implies that yellow feathers contain a more homogeneous mixture of pigments compared with red feathers. Cooke *et al.* [13] provided evidence that pigment diversity differs between red and yellow feathers. We provide evidence that chromophore diversity also differs between red and yellow feathers. The presence of the C_20_ psittacofulvin in red feathers, and perhaps also the presence of aldehyde groups in the red psittacofulvins and changes in total pigment concentration effectively result in a heterogeneous mixture of chromophores in red feather barbs compared with the singular chromophore behaviour of the yellow feather we studied here.

### 4.3. Summary and future directions

Yellow colours in parrot feathers contain one dominant chromophore and not the multiple chromophores found in red feather barbs, despite the expectation that both red and yellow feathers contain mixtures of non-isomeric pigments. The psittacofulvin pigments in red feathers may, therefore, be structurally organized by molecule–molecule interactions, resulting in a heterogeneous mixture of chromophore behaviours. The multiple chromophores we observe in red feather barbs absorb over a range of wavelengths and could be the basis of a colour-tuning mechanism. Here we surmise from published data and our own observations that parrots produce different mixtures of psittacofulvin pigments during feather growth, and release these pigments into the growing feather at different times (and at different concentrations) to produce magenta, red, orange and yellow coloration. Major differences in hue are associated with differences in the organization of pigments in the feather barb, and minor differences in hue are linked to molecular associations that may be induced by differences in pigment concentration.

Our study shows the utility of Raman frequency dispersion for analysing chromophore mixtures in animal integuments. This frequency dispersion technique could provide valuable natural history information for other pigment systems where molecule–molecule interactions have been proposed [44,45]. Describing how the structures of psittacofulvins result in yellow and red feather regions will provide deeper insight into the evolutionary origins of parrot plumage coloration.

## Supplementary Material

Supporting data

## Supplementary Material

Figure S1

## Data accessibility

Electronic supplementary material, tables S1–S5.

## Appendix A. Specimen and analysis details for 26 parrot study skins housed in the Division of Birds, National Museum of Natural History, Smithsonian Institution (Washington, DC, USA)

**Table:**

species (abbreviation)	common name	specimen number (USNM)	analysed feather location	analysed feather colour
*Agapornis taranta* (Ata)	black-winged lovebird	569413	crown	red
*Alisterus scapularis* (Asc)	Australian king parrot	632144	plucked contour, location unknown	red
*Ara ararauna* (Aar)	blue and yellow macaw	not recorded	breast	yellow-orange
*Ara macao* (Ama)	scarlet macaw	112234	tail	red
*Aratinga canicularis* (Aca)	orange-fronted parakeet	646903	crown	orange
*Aratinga pertinax* (Ape)	brown-throated parakeet	632380	cheek	yellow-orange
*Cacatua galerita* (Cga)	sulfur-crested cockatoo	not recorded	crest	yellow
*Cacatua haematuropygia* (Cha)	red-vented cockatoo	578568	under tail covert	orange
*Cacatua moluccensis* (Cmo)	salmon-crested cockatoo	599494	crest	orange
*Charmosyna papou* (Cpa)	Papuan lorikeet	611650	dorsal contour	red
*Eolophus roseicapilla* (Ero)	galah	632234	flank	magenta
*Guarouba guarouba* (Ggu)	golden parakeet	622396	tail	yellow
*Loriculus philippensis* (Lph)	Philippine hanging parrot	201837	dorsal contour, above tail	red-orange
*Neophema chrysogaster* (Nch)	orange-bellied parrot	405781	ventral contour	orange
*Nestor notabilis* (Nno)	kea	not recorded	under wing	orange
*Nymphicus hollandicus* (Nho)	cockatiel	602201	cheek	orange
*Pionites melanocephala* (Pme)	black-headed parrot	632411	two plucked contours, location unknown	yellow-orange and orange-red
*Pionopsitta caica* (Pca)	Caica parrot	637228	wing	yellow
*Pionopsitta haematotis* (Pha)	brown-hooded parrot	562541	wing	red
*Pionus fuscus* (Pfu)	dusky parrot	637146	plucked contour, location unknown	red
*Platycercus eximius* (Pex)	eastern rosella	614459	under tail covert	red
*Poicephalus rueppelli* (Pru)	Rüppell's parrot	642638	shoulder	yellow
*Polytelis alexandrae* (Pal)	princess parrot	405761	tail	pink
*Psittaculirostris edwardsii* (Ped)	Edwards’ fig parrot	611652	flank	orange
*Strigops habroptilus* (Sha)	kakapo	uncertain^a^	loose contour, location unknown	yellow

^a^Feather was loose in the drawer that contained only kakapo study skins.

## Appendix B

The energy of vibrational modes can be related to bond force constant (i.e. *k*_C-C_ and *k*_C=C_) through a simple harmonic oscillator (equation B 1):

B 1 $$\tilde{\nu }=\frac{1}{2\pi c}\sqrt{\frac{k}{\mu }},$$

where $\tilde{\nu }$ is vibrational energy, *c* is the speed of light, *k* is the force constant for the bond of interest and *μ* is the reduced mass of the bonded units. As the excitation wavelength is varied, resulting in a change of the observed vibrational energy, there must also be a change of either force constant or reduced mass or both. The effective conjugation length of aromatic chromophores is well known to have a consequence on the absorption *λ*_max_, established by the particle in a box theory. Changes in conjugation length of psittacofulvin pigments would also be expected to perturb $\tilde{\nu }(\text{C−C})$ and $\tilde{\nu }(\text{C=C})$ vibrational energies, as seen experimentally.
